# Norwegian reference values for the Short Physical Performance Battery (SPPB): the Tromsø Study

**DOI:** 10.1186/s12877-019-1234-8

**Published:** 2019-08-08

**Authors:** Astrid Bergland, Bjørn Heine Strand

**Affiliations:** 1 Department of Physiotherapy, Faculty of Health Sciences, Oslo Metropolitan University, Pilestredet, St. Olavs Plass, P.O. Box 4, 0130 Oslo, Norway; 2 Department of Chronic Diseases and Ageing, Norwegian Institute of Public Health, Oslo, Norway; 3 Department of Community Medicine, Institute of Health and Society, University of Oslo, Oslo, Norway; 4 Norwegian National Advisory Unit on Ageing and Health, Tønsberg, Norway

**Keywords:** Physical function, Mobility, Performance based, Reference values

## Abstract

**Background:**

The Short Physical Performance Battery (SPPB) is a common well-established instrument to measure physical performance. It involves a timed 4-m walk, timed repeated chair sit-to-stand test, and 10-s balance tests (side-by-side, semi-tandem, and full-tandem). We aimed to establish reference values for community-dwelling Norwegian adults aged 40 years or older in terms of (1) the total score; (2) the three subtest scores; and (3) the time to complete the repeated chair sit-to-stand test and the walking speed. Additionally, we explored floor and ceiling effects for the SPPB.

**Methods:**

The study population comprised home dwellers aged 40 years or more who participated in the 7th wave of the Tromsø study. A sample of 7474 participants (53.2% women) completed the SPPB. Crude mean values and standard deviations (SD) were evaluated according to sex and age group. Mean values at specific ages were then estimated using linear regression, along with corresponding 95% confidence intervals. Additionally, quantile regression was used to estimate age-specific percentiles (5th, 10th, 25th, 50th, 75th, 90th, and 95th percentiles).

**Results:**

Considerable variability in SPPB scores was observed. The mean SPPB total score of the entire sample was 11.4 (SD 1.3) points. On average, the SPPB total score was 0.28 points greater in men than in women (*p* < 0.001). Significant sex differences were observed in all five age groups (40–49, 50–59, 60–69, 70–74, 75–79, and 80+ years). The main decline in the physical function occurred in the mid-sixties, with a slightly earlier decline in women than in men. Ceiling effects were observed in all age groups.

**Conclusions:**

The present study provides comprehensive, up-to-date normative values for SPPB measures in community-dwelling Norwegians aged at least 40 years that may be used to interpret the results of studies evaluating and establishing appropriate treatment goals. Because of ceiling effects, the SPPB has important limitations for the assessment of physical functioning across the full spectrum of the community-dwelling adults aged 40+ years. Furthermore, we conclude that performance on the SPPB should be reported in terms of the total sum score and registered time to complete the repeated chair sit-to stand test and timed 4-m walk test.

## Background

Physical function is a strong measure of biological age and a biomarker for health and quality of life in older people [1–4]. The assessment of physical function among older adults is of importance, as the early detection of functional decline renders it possible to intervene and reverse or prevent further physical function decline and the possible loss of independence [5]. Furthermore, physical performance assessment as an outcome measure is a vital component in studies comparing groups or evaluating the effect of different interventions on physical function [6, 7].

The Short Physical Performance Battery (SPPB) is a well-established instrument for the measurement of physical performance, commonly used among community-dwelling adults, nursing home residents, and hospitalized patients [1, 5–12]. The SPPB involves a timed 4-m walk at the participant’s normal pace, a timed repeated chair sit-to-stand test, and 10-s balance tests, with feet side-by-side, semi-tandem, and full-tandem. Low SPPB scores have been shown to predict poor outcomes, such as falls, mobility loss, disability, hospitalization, a longer hospital stay, nursing home admission, and death [1, 6–8, 11, 13–16]. Furthermore, previous research suggests that the SPPB can detect the early stages of frailty [17], and that a total score ≤ 9 points can distinguish frail from non-frail individuals [18].

As a performance-based measure of physical function, the SPPB has many advantages. The SPPB only takes a few minutes to complete, requires little training to administer, and uses simple equipment. Additionally, the results can be quantified by scores, and it is reproducible and sensitive to changes in functionality through time [18]. Previous systematic reviews that evaluated the psychometric properties of various physical performance instruments have concluded that the SPPB is a reliable and valid tool for measuring lower limb strength in the elderly community [5, 19, 20]. Therefore, the SPPB is considered a good measure for cross-cultural comparisons of physical performance in elderly individuals [18]. The Norwegian translation of the SPPB [21] has shown high reliability in elderly people with and without dementia, living at home or in nursing homes [22].

To be meaningful, test scores must have an empirical frame of reference. Reference or normative (used as synonyms in this paper) data provide this empirical context and represent the range of performance for a particular test in a particular group of individuals. Normative data provide a numerical description of test performance in a well-defined sample group [23]. This group is considered the ‘gold standard’ against which an individual‘s test performance is compared and contrasted [24]. In particular, percentiles, which indicate a person’s relative position in the group for the ability/characteristics tested, provide a useful way of identifying individuals with performance significantly below the level expected for their age and background [23]. One consideration in choosing an appropriate normative dataset is the dataset’s sample size [24]. Furthermore, the use of reference data for a specific population is recommended for a more meaningful interpretation of physical function test results [25]. Thus, the optimal reference values for a physical function test must consider differences in sex and age [26].

Despite the critical importance of having access to normative data to facilitate the clinical interpretation of test findings, there are relative few large-scale normative reports in the literature [24]. As yet, there are no published reference values for the SPPB (in terms of the total score) based on a large sample of individuals aged 40+ years. Thus, we aimed to establish reference values, stratified by sex and age (as recommended by Steffen et al. [27]), for community-dwelling Norwegian adults aged 40 years or older in terms of (1) the SPPB total score; (2) the scores of the three subtests (balance, walking speed, and repeated chair sit-to stand); and (3) the walking speed test (in m/s) and time (in seconds) to complete 5 chair stands in the chair sit-to-stand test. Additionally, we aimed to explore floor and ceiling effects in these measures.

## Methods

### Study population

Participants comprised men and women aged 40 years or more who participated in the 7th wave of the Tromsø study [28]. The Tromsø study is a multipurpose population-based health examination study, initiated in 1974, with study waves repeated in 1979, 1986, 1994, 2001, 2008, and 2015. In the current analyses, the sample was restricted to those participating in the wave initiated in 2015. All Tromsø study participants aged 40 years or more were invited to complete phase-one of the Tromsø study (*n* = 32,591), and a random subset of 40% were invited to complete the phase-two examination, which comprised a more thorough clinical examination and included physical function testing. Some Tromsø study participants were invited to complete all phase-two subtests, while most were invited to only some of the tests. The Regional Committee of Research Ethics approved the study (2016/389), and written informed consent was obtained from all participants in the Tromsø Study.

We included individuals who completed the SPPB with non-missing values for all subtests. Among the 9324 individuals invited to SPPB testing, 7866 participated; of these 7763 had non-missing data (Table 1). However, we excluded those who performed the tests without shoes (*n* = 279) and those who required assistance or had short-term leg injuries (*n* = 10). The use of walking aids was allowed, and were used by 31 participants (crutches/cane: *n* = 27; walker, *n* = 4). Thus, our final study population comprised 7474 participants (53.2% women).

**Table 1:** Background characteristics and SPPB score distribution according to sex and age, *n* = 7474

Age (years)	40–59	60–69	70–74	75–79	80+
Men					
Mean age (SD)*	50.3 (5.9)	64.6 (2.9)	71.8 (1.4)	76.8 (1.3)	81.6 (1.3)
Education, n (%)					
Basic	148 (12)	515 (33)	299 (49)	194 (58)	96 (65)
Middle	351 (28)	426 (28)	152 (25)	67 (20)	34 (23)
Tertiary	745 (60)	601 (39)	159 (26)	72 (22)	17 (12)
Missing (%)	0%	1%	3%	6%	11%
Mean height, cm (SD)	179.1 (6.6)	176.8 (6.6)	175.8 (6.1)	173.7 (6.4)	173.3 (6.4)
Mean weight, kg (SD)	89.6 (14.6)	87.1 (13.3)	86.1 (12.6)	82.8 (12.4)	79.6 (11.5)
Mean BMI, kg/m^2^ (SD)	27.9 (4.2)	27.9 (3.9)	27.8 (3.6)	27.4 (3.7)	26.5 (3.3)
SPPB total*					
0–3, %	0	0	0	0	2
4–6, %	0	0	0	1	5
7–9, %	1	3	7	15	21
10–12, %	99	97	92	84	72
Mean SPPB score (SD)	11.9 (0.6)	11.7 (0.8)	11.4 (1.1)	10.8 (1.5)	10.1 (2.3)
Women					
Mean age (SD)*	50.6 (5.9)	64.6 (2.9)	71.8 (1.4)	76.8 (1.4)	81.7 (1.5)
Education, n					
Basic	174 (17)	397 (28)	157 (31)	96 (32)	67 (47)
Middle	322 (31)	405 (28)	151 (30)	97 (32)	37 (26)
Tertiary	544 (52)	639 (44)	197 (39)	106 (35)	38 (27)
Missing (%)	1%	2%	3%	6%	14%
Mean height, cm (SD)	165.6 (6.3)	163.6 (6.1)	162.1 (5.8)	160.5 (6.0)	158.7 (5.8)
Mean weight, kg (SD)	72.9 (13.8)	71.9 (12.6)	73.3 (13.8)	69.3 (12.5)	67.7 (12.3)
Mean BMI, kg/m^2^ (SD)	26.6 (5.0)	26.9 (4.6)	27.9 (5.1)	26.9 (4.7)	26.9 (4.6)
SPPB total*					
0–3, %	0	0	0	1	3
4–6, %	0	0	1	3	9
7–9, %	2	5	14	24	27
10–12, %	98	95	84	72	61
Mean SPPB score (SD)	11.8 (0.6)	11.4 (1.1)	10.8 (1.6)	10.2 (1.9)	9.5 (2.5)
Mean age (SD)*	50.6 (5.9)	64.6 (2.9)	71.8 (1.4)	76.8 (1.4)	81.7 (1.5)

*Age distribution did not differ significantly between the sexes (Pearson Chi squared test, *p* = 0.22) *Cells with 4 or less individuals are set to ‘-‘

### SPPB procedures

From April 20th 2015 to October 26th 2016, experienced clinical evaluators (physiotherapists and trained nurses) assessed the SPPB using standardized methodologies for the instructions, positioning, and scoring. Seven different evaluators rotated during this time period, each spending 1 week at a time in the SPPB station.

The standing balance tests included tandem, semi-tandem and side-by-side standing, and the participants were timed until they moved or 10 s had elapsed. To assess walking speed, the participants were twice asked to walk 4 m at their regular pace. For the repeated chair sit-to-stand test, a pre-test was performed; the participants were asked to fold their arms across their chest (i.e. the armrests were not used) and stand up from the chair. If the pre-test was successful, the participants were asked to perform five chair stands as quickly as possible. They were timed (in seconds) from the initial sitting position to the final standing position at the fifth stand. Each of the three subtests (balance, walking speed and repeated chair sit-to-stand test) of the SPPB was scored from 0 to 4, and summed for a total score ranging from 0 to 12, with higher scores reflecting better function. In addition, the walking speed (meters/second) was calculated as 4/ (the fastest time [in seconds] of the two walking speed trials). Four total SPPB score categories (0–3, 4–6, 7–9, 10–12) according to the cut-points provided by Guralnik and colleagues in their original work [6] is used.

### Covariates

Age and sex data were obtained from the Tromsø study registry. All participants were asked about their highest completed level of education. The education level was classified into three categories: second level, first stage (elementary and/or primary school); second level, second stage (high school); and third level (college or university). Height and weight were measured in light clothing without shoes. Body mass index (BMI) was calculated as the weight in kilograms divided by the height in meters squared (kg/m^2^).

### Statistical analysis

Crude mean values and standard deviations (SD) stratified by sex and age groups were first determined. Mean values at specific ages were then estimated, along with corresponding 95% confidence intervals, in linear regression analyses. Next, quantile regression was used to estimate age-specific percentiles (5th, 10th, 25th, 50th, 75th, 90th, and 95th percentiles). In both regression settings, age was included as a restricted cubic spline with 4 knots at default knot locations (ages 44, 61, 68, and 79 years). Models were run separately for men and women. The SD was estimated from the regression model and reflects the standard error of the forecasted value, which corresponds to the SD and is a measure of variation in the actual values. Additionally, we fitted 95% prediction intervals for the walking speed and repeated chair sit-to-stand test to indicate the distribution of the actual individual values. Sex-specific normative values for the SPPB, chair sit-to-stand test and walking speed at five-year age intervals (40, 45, ..., 85 years) were then predicted post hoc from the fitted regression models. Finally, floor and ceiling effects were considered as present when more than 20% of the respondents achieved the lowest or highest possible score [29, 30].

## Results

**Fig. 1:**
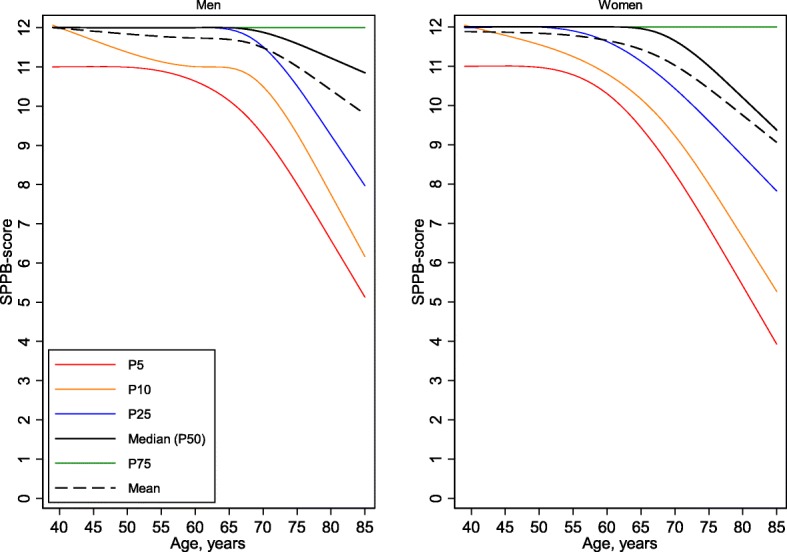
SPPB total score by age and sex. Percentiles (5th, 10th, 25th, 50th, 75th) and the mean value are shown

**Fig. 2:**
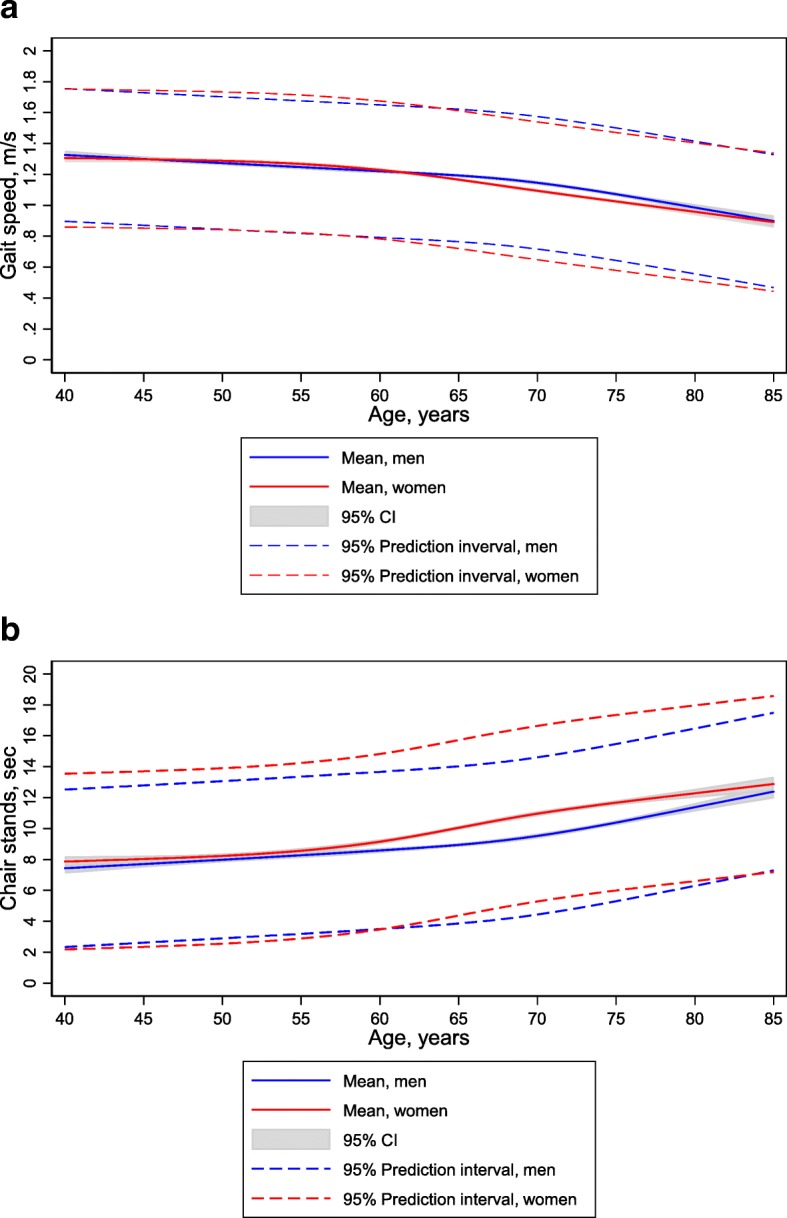
**a** Walking speed (m/sec) by age and sex. **b** Chair sit-to- stand test (sec) by age and sex. Mean values with corresponding 95% confidence intervals (CIs) and 95% prediction interval (prediction interval is indicative of the distribution of the actual individual values) are shown

Demographic (sex, age, and level of education) and anthropometric data (height, weight, and BMI) are summarized in Table 1. The mean age of the total sample (7474 participants; 53.2% women) was 63.2 years (SD, 10.4 years; range, 40–85 years). In general, decreased function with increased age was observed (Figs. 1, 2a and b).

The mean total SPPB score of the total sample was 11.4 points (SD, 1.3; range, 0–12). The mean of the total SPPB score, as well as the distribution of three SPPB classes (total SPPB ≤6, 7–9, and > 9 points), are shown according to sex and age group in Table 1. On average, the total SPPB score was 0.28 points greater in men than in women (*p* < 0.001), with significant sex differences in all five age groups (Table 1). Age-specific percentile reference data for the total SPPB score in men and women are shown in Fig. 1. The mean and median of the total SPPB score were approximately 12 points (at the maximum) until the age of 70 years in men and 65 years in women; thereafter, there was a steep decline with increased age. Observed ceiling effect for men, defined as more than 20% with the maximum score for the age groups of 40–49; 50–59; 60–69, 70–79 was 80+ was 91, 78, 64, 47, and 36%, respectively. Furthermore observed *ceiling effect* for women for the age groups of 40–49; 50–59; 60–69, 70–79 was 80+ years was 88, 65, 44, 31, and 23%, respectively.

The distribution of scores for each of the subtests is shown in Table 2. The mean balance, walking speed, repeated chair sit-to-stand scores of the total sample were 3.85 (SD, 0.50), 3.90 (SD, 0.36), and 3.63 (SD, 0.78), respectively. The mean walking speed (meter/second) with 95% confidence bands, as well as the 5th and 95th percentiles to further illustrate the range, is shown in Fig. 2a. The decline in walking speed with age was similar across sex until the age of 60–65 years; starting at this age, the decline in women was greater than that in men. However, men had a steep decline at approximately 75 years of age, resulting in similar walking speeds for men and women at 80–85 years of age. Furthermore, performance in the repeated chair sit-to-stand test was similar between men and women until approximately 60 years of age and after 80 years of age, with women performing significantly worse than men from 60 to 80 years of age (Fig. 2b). Additionally, walking speed was significantly greater in men than in women for the age groups of 65–69 and 70–74 years, but not for the other age groups (Table 3). The mean time (in seconds) to complete the repeated chair sit-to-stand test is shown according to sex and age group in Table 4.

**Table 2:** Distribution of SPPB subtest scores according to sex and age. *n* = 7474

Sex, age (years), and SPPB subtest	Mean score (SD)	Subtest score				
		0	1	2	3	4
Men, *n* = 3501						
40–59, *n* = 1044						
Balance	3.98 (0.18)	–	–	–	9 (1)	1032 (99)
Walking speed	3.99 (0.12)	–	–	–	12 (1)	1031 (99)
Chair sit-to-stand test	3.89 (0.45)	–	–	15 (1)	59 (6)	962 (93)
60–69, *n* = 1460						
Balance	3.91 (0.36)	–	–	27 (2)	65 (4)	1366 (94)
Walking speed	3.96 (0.21)	–	–	5 (0)	49 (3)	1406 (96)
Chair sit-to-stand test	3.78 (0.60)	7 (0)	17 (1)	41 (3)	160 (11)	1235 (85)
70–74, *n* = 519						
Balance	3.83 (0.48)	–	–	20 (4)	46 (9)	452 (87)
Walking speed	3.90 (0.35)	–	–	8 (2)	35 (7)	476 (92)
Chair sit-to-stand test	3.65 (0.72)	4 (1)	12 (2)	17 (3)	96 (18)	390 (75)
75–79, *n* = 319						
Balance	3.69 (0.69)	–	5 (2)	20 (6)	41 (13)	252 (79)
Walking speed	3.76 (0.49)	–	–	10 (3)	55 (17)	254 (80)
Chair sit-to-stand test	3.39 (0.88)	4 (1)	9 (3)	34 (11)	83 (26)	189 (59)
80+, *n* = 159						
Balance	3.45 (0.93)	–	–	21 (14)	25 (16)	107 (70)
Walking speed	3.64 (0.71)	–	–	7 (4)	32 (21)	117 (75)
Chair sit-to-stand test	3.04 (1.19)	6 (4)	15 (9)	28 (18)	27 (17)	83 (52)
Women, *n* = 3973						
40–59, *n* = 1252						
Balance	3.98 (0.20)	–	–	6 (0)	13 (1)	1232 (98)
Walking speed	3.98 (0.15)	–	–	–	20 (2)	1230 (98)
Chair sit-to-stand test	3.85 (0.50)	6 (0)	8 (1)	20 (1)	94 (7)	1224 (91)
60–69, *n* = 1566						
Balance	3.89 (0.40)	–	–	25 (2)	113 (7)	1424 (91)
Walking speed	3.92 (0.31)	–	–	10 (1)	88 (6)	1465 (94)
Chair sit-to-stand test	3.60 (0.75)	10 (1)	41 (3)	74 (5)	310 (20)	1131 (72)
70–74, *n* = 630						
Balance	3.71 (0.65)	–	–	41 (7)	78 (13)	505 (81)
Walking speed	3.82 (0.47)	–	–	15 (2)	75 (12)	538 (86)
Chair sit-to-stand test	3.27 (0.99)	13 (2)	31 (5)	73 (12)	168 (27)	345 (55)
75–79, *n* = 354						
Balance	3.52 (0.86)	5 (1)	8 (2)	32 (9)	63 (18)	246 (69)
Walking speed	3.65 (0.64)	–	–	10 (3)	88 (25)	251 (72)
Chair sit-to-stand test	3.08 (1.11)	15 (4)	22 (6)	48 (14)	105 (30)	164 (46)
80+, *n* = 171						
Balance	3.19 (1.05)	4 (2)	7 (4)	36 (21)	29 (17)	95 (56)
Walking speed	3.55 (0.78)	–	–	16 (10)	32 (19)	119 (71)
Chair sit-to-stand test	2.73 (1.29)	14 (8)	20 (12)	26 (15)	49 (29)	62 (36)

SPPB subtest scores ranged 0–4. Data are reported as number of participants (%)

**Table 3:** SPPB subtest: Walking speed, m/s by sex and age

Age (years)	Men		Women		Gender-diff, *p*-value*
	N	Mean (SD)	N	Mean (SD)	
40–44	213	1.31 (0.22)	263	1.31 (0.23)	0.71
45–49	260	1.29 (0.20)	283	1.29 (0.21)	0.79
50–54	250	1.25 (0.20)	306	1.29 (0.23)	0.08
55–59	321	1.24 (0.21)	400	1.26 (0.23)	0.32
60–64	719	1.21 (0.22)	761	1.20 (0.22)	0.40
65–69	741	1.18 (0.21)	804	1.13 (0.23)	< 0.01
70–74	519	1.12 (0.23)	629	1.08 (0.24)	< 0.01
75–79	319	1.03 (0.25)	352	1.00 (0.24)	0.07
80+	157	0.97 (0.21)	170	0.94 (0.22)	0.17

*t-test

**Table 4:** SPPB subtest: Repeated chair sit-to stand test, seconds by sex and age

Age (years)	Men		Women		Gender-diff, *p*-value*
	N	Mean (SD)	N	Mean (SD)	
40–44	213	7.5 (2.1)	263	7.8 (2.0)	0.11
45–49	259	7.9 (1.9)	282	8.1 (2.5)	0.26
50–54	247	8.0 (2.4)	303	8.6 (2.4)	< 0.01
55–59	320	8.4 (2.3)	398	8.7 (2.3)	0.07
60–64	715	8.7 (2.4)	758	9.4 (2.7)	< 0.01
65–69	738	9.2 (2.8)	798	10.5 (3.2)	< 0.01
70–74	515	9.7 (2.7)	616	11.3 (3.3)	< 0.01
75–79	315	10.7 (2.9)	339	11.7 (3.2)	< 0.01
80+	153	11.9 (3.8)	157	12.6 (4.1)	0.11

*t-test. There were 83 individuals that failed on the pre-test and did not do the repeated chair sit-to- stand test

Among men, 91, 78, 64, 47, and 36% had a total SPPB score of 12 points in the age groups of 40–49, 50–59, 60–69, 70–74, 75–79, and 80+ years, respectively. The corresponding rates for women were 88, 65, 44, 31, and 23%, respectively. No floor effects were observed for the total SPPB score. For the balancing, walking speed, and repeated chair sit-to-stand tests, low scores (0–2 points) were observed in 14, 4, and 31% of those in the oldest male age group (80–85 years). The corresponding values for women aged 80–85 years were 27, 16 and 60%, respectively. Ceiling effects were observed in the youngest age groups for all three subtests; however, no floor effects were observed.

Sex- and age-specific percentile reference values for the SPPB sub tests walking speed and chair sit-to stand test are presented in Tables 5, 6 and 7.

**Table 5:** Normative values for total SPPB score

Age (years)	Mean	SD	P5	P10	P25	P50	P75	P90	P95
Men									
40	11.99	1.00	11	12	12	12	12	12	12
45	11.91	1.00	11	11.7	12	12	12	12	12
50	11.84	1.00	11	11.4	12	12	12	12	12
55	11.78	1.00	10.9	11.1	12	12	12	12	12
60	11.74	1.00	10.6	11.0	12	12	12	12	12
65	11.70	1.00	10.1	11.0	12	12	12	12	12
70	11.49	1.00	9.3	10.5	11.5	11.9	12	12	12
75	11.01	1.00	8.0	9.3	10.5	11.6	12	12	12
80	10.41	1.00	6.6	7.7	9.3	11.2	12	12	12
85	9.80	1.00	5.1	6.2	8.0	10.8	12	12	12
Women									
40	11.88	1.24	11	12	12	12	12	12	12
45	11.86	1.24	11.0	11.8	12	12	12	12	12
50	11.83	1.24	11	11.6	12	12	12	12	12
55	11.78	1.24	10.8	11.2	11.9	12	12	12	12
60	11.65	1.24	10.3	10.8	11.6	12	12	12	12
65	11.43	1.24	9.4	10.2	11.1	12	12	12	12
70	11.02	1.24	8.3	9.2	10.4	11.7	12	12	12
75	10.43	1.24	6.9	8.0	9.6	11	12	12	12
80	9.75	1.24	5.4	6.6	8.7	10.2	12	12	12
85	9.06	1.24	3.9	5.3	7.8	9.3	12	12	12

**Table 6:** Normative values for the SPPB walking speed test (m/s)

Age (years)	Normative values of SPPB-sub-scale: Walking speed m/s								
	Mean	SD	P5	P10	P25	P50	P75	P90	P95
Men									
40	1.32	0.22	1.04	1.08	1.18	1.29	1.46	1.60	1.71
45	1.30	0.22	1.00	1.05	1.16	1.28	1.43	1.57	1.67
50	1.27	0.22	0.95	1.01	1.13	1.26	1.40	1.54	1.63
55	1.25	0.22	0.91	0.98	1.11	1.24	1.37	1.52	1.60
60	1.22	0.22	0.88	0.95	1.08	1.22	1.35	1.50	1.58
65	1.19	0.22	0.85	0.92	1.04	1.18	1.33	1.47	1.57
70	1.15	0.22	0.81	0.88	0.99	1.13	1.29	1.44	1.54
75	1.07	0.22	0.74	0.81	0.91	1.05	1.22	1.37	1.46
80	0.99	0.22	0.65	0.72	0.83	0.97	1.14	1.30	1.35
85	0.90	0.22	0.56	0.64	0.74	0.89	1.06	1.22	1.25
Women									
40	1.31	0.23	0.95	1.04	1.15	1.28	1.46	1.58	1.64
45	1.30	0.23	0.95	1.03	1.15	1.28	1.44	1.58	1.64
50	1.29	0.23	0.95	1.02	1.14	1.27	1.44	1.57	1.65
55	1.27	0.23	0.93	1.00	1.12	1.26	1.41	1.55	1.64
60	1.23	0.23	0.89	0.95	1.07	1.22	1.37	1.52	1.61
65	1.17	0.23	0.82	0.88	1.00	1.16	1.31	1.46	1.56
70	1.09	0.23	0.74	0.81	0.92	1.09	1.24	1.39	1.48
75	1.02	0.23	0.67	0.73	0.85	1.02	1.18	1.32	1.41
80	0.96	0.23	0.60	0.66	0.79	0.96	1.12	1.25	1.33
85	0.89	0.23	0.54	0.59	0.73	0.90	1.06	1.17	1.25

**Table 7:** Normative values for the SPPB repeated chair sit-to-stand test (seconds)

Age	Normative values of SPPB-sub-scale: Repeated chair sit-to-stand test, seconds								
	Mean	SD	P5	P10	P25	P50	P75	P90	P95
Men									
40	7.4	2.6	4.8	5.3	6.0	7.3	8.5	9.8	10.7
45	7.7	2.6	5.0	5.4	6.2	7.5	8.8	10.3	11.2
50	8.0	2.6	5.1	5.5	6.4	7.7	9.1	10.8	11.6
55	8.3	2.6	5.2	5.7	6.6	8.0	9.4	11.2	12.0
60	8.6	2.6	5.5	5.9	6.9	8.3	9.8	11.5	12.5
65	8.9	2.6	5.7	6.2	7.3	8.7	10.2	11.8	12.9
70	9.5	2.6	6.1	6.6	7.7	9.3	10.9	12.5	13.9
75	10.4	2.6	6.4	7.0	8.3	10.0	12.1	14.0	15.4
80	11.4	2.6	6.6	7.5	8.9	10.7	13.5	15.8	17.2
85	12.4	2.6	6.9	7.9	9.6	11.5	14.9	17.7	19.0
Women									
40	7.9	2.9	5.0	5.6	6.5	7.5	8.9	10.5	11.7
45	8.0	2.9	5.1	5.6	6.6	7.7	9.1	10.7	11.8
50	8.2	2.9	5.2	5.7	6.7	7.9	9.4	10.9	12.0
55	8.6	2.9	5.4	5.8	6.9	8.3	9.8	11.3	12.4
60	9.1	2.9	5.8	6.3	7.4	8.9	10.5	12.1	13.2
65	10.0	2.9	6.4	7.0	8.2	10.0	11.4	13.3	14.6
70	10.9	2.9	7.0	7.7	8.9	10.5	12.4	14.6	16.1
75	11.7	2.9	7.4	8.2	9.5	11.2	13.1	15.8	17.4
80	12.3	2.9	7.6	8.5	10.0	11.7	13.7	16.8	18.5
85	12.9	2.9	7.9	9.0	10.5	12.3	14.3	17.8	19.7

Tables 5, 6, 7: Values for the percentiles were estimated from quantile regression analyses, while the mean (SD) was estimated from a linear regression model. In both regression settings, age was included as a restricted cubic spline with 4 knots at default knot locations (age: 44, 61, 68, and 79 years). Models were run separately for men and women. SD was estimated from the regression model and is the standard error of the forecast. P5, P10, P25, P50, P75, P90, P95; the 5th, 10th, 25th, 50th, 75th, 90th, and 95th percentile, respectively.

## Discussion

To the best of our knowledge, the present study is the first to provide sex-specific reference values for the SPPB total score, as well as for the three subtests included in the SPPB, in community-dwelling adults aged at least 40 years. There was considerable variability in the SPPB total score among individuals age 40+ years living at home. Furthermore, the present study results demonstrate that the main decline in physical function occurs in the mid-sixties, with a slightly earlier decline in women than in men.

An appropriate measuring instrument should have minimal floor and ceiling effects for the intended purpose and population [31]. The present study showed a considerable ceiling effect using for the SPPB total and subtest scores, since more than 20% of the respondents achieved the lowest or highest possible score [29, 30]. Consistent with the present results, ceiling effects for physical performance measurement instruments in higher-functioning community-dwelling older adults aged ≥60 years have been observed by other researchers [31, 32]. Furthermore, the detection of ceiling effects in the youngest age groups for the SPPB, scored in terms of points, is not surprising [7, 33]. However, ceiling effects for physical performance measurement instruments do not only hamper the detection of early balance deficits, but also prevent the detection of intervention-related changes over time in higher-functioning older adults [7, 32–34]. When a measure is used to capture change, high baseline scores and ceiling effects limit the ability to detect improvement between two assessments, posing a serious concern for type II errors in clinical trials. Even when the more serious risk of type II errors does not occur, outcome measures with limited sensitivity to change may falsely diminish the overall magnitude of the intervention effect. This suggests that reporting the performance on the subtests of the SPPB as the time to complete a 3-m or 4-m walk and the time to rise from a chair five times in the repeated chair sit-to-stand test might be better for high-functioning adults aged 40–80 years.

The present study demonstrated a significant trend toward age-related functional decline, with some differences between men and women, consistent with previous studies [35]. Furthermore, a previous meta-analysis, which clearly highlighted an effect of age on walking speed [36], reported mean walking speeds stratified by sex and age-group (in 10-year intervals) that correspond quite well to the present results. The present data on walking speed in men and women at different ages also correspond well to those in the review of reference values for standardized tests of walking speed by Salbach et al. [37] and the study by Callisaya et al. [38], which randomly selected participants from the Southern Tasmanian electoral roll (*n* = 223). Additionally, Thaweewannakij et al. [35] described reference values for the comfortable walking speed in elderly people, aged 60–90 years, who were well functioning and dwelling in the community. The speed varied from 0.88 to 1.48 m/s, which corresponds well to the present results, even though the walking distances differed between the studies. However, our participants performed better on the repeated chair sit-to- stand test than did the participants in the study by Thaweewannakij et al. [35], with times ranging from 12.9 s in the age group of 60–69 years to 17.1 s in women aged 80 or more (see Table 4). A walking speed < 0.6 m/s on the 4-m test has been used as to identify persons at high risk for being hospitalized with deteriorating health and physical function [12]. All of our participants had a walking speed >0.6 m/s, which differs from the rate of 8.1% reported in other studies [39]. As Da Câmara et al. [18] reported that 9 points on the SPPB discriminates between frail and non-frail older adults, approximately 20% of men and women aged 75 years or more in our study population could be classified as frail.

### Strengths and limitations of the study

One strength of the present study is its use of a performance-based physical function assessment that was previously tested for validity and reliability [5]. Furthermore, before the study was initiated, the testers completed a training programme to ensure high inter-rater test reliability. Additionally, the current study has a high degree of generalizability as it recruited from the general population. However, the study focused only on community-dwelling older people, omitting those living in institutions, and it remains unclear whether the present findings are generalizable beyond Norway. Additionally, legal restrictions hamper detailed comparisons between participants and non-participants [28]. In general, studies of the two first waves (Tromsø 4 and 6) revealed differences in age and marital status between participants and non-participants; non-participants were younger and more likely to be single [28].

## Conclusions

The present study is the first to provide comprehensive, up-to-date normative values for SPPB measures in community-dwelling individuals aged at least 40 years and living in Norway. Up-to-date population-specific normative values are essential in enabling clinicians to better evaluate patient performance relative to that for the general population community-living older adults and determine the appropriate intervention/management. Because of ceiling effects, the SPPB has limitations in the assessment of physical functioning across the full spectrum of community-dwelling adults aged 40+ years that should be considered. Finally, we conclude that performance on the SPPB should be reported in terms of the total score, as well as the time to complete the repeated chair sit-to-stand test and the walking speed test. The present data may be used to interpret the results of studies evaluating and establishing appropriate treatment goals.

## Data Availability

The data supporting the conclusions of this article are available at www.tromsoundersokelsen.no

## Abbreviations

BMI: Body mass index
CI: Confidence interval
SD: Standard deviation
SD: Standard deviations
SPPB: Short Physical Performance Battery
